# A novel method of transcriptome interpretation reveals a quantitative suppressive effect on tomato immune signaling by two domains in a single pathogen effector protein

**DOI:** 10.1186/s12864-016-2534-4

**Published:** 2016-03-14

**Authors:** Jay N. Worley, Marina A. Pombo, Yi Zheng, Diane M. Dunham, Christopher R. Myers, Zhangjun Fei, Gregory B. Martin

**Affiliations:** Boyce Thompson Institute for Plant Research, Ithaca, NY 14853-1801 USA; Institute of Biotechnology, Cornell University, Ithaca, NY 14853-1801 USA; Section of Plant Pathology and Plant-Microbe Biology, School of Integrative Plant Science, Cornell University, Ithaca, NY 14853-1801 USA

**Keywords:** RNA-Seq, Pattern-triggered immunity, Plant immunity, Type III effectors, *Pseudomonas syringae*

## Abstract

**Background:**

Effector proteins are translocated into host cells by plant-pathogens to undermine pattern-triggered immunity (PTI), the plant response to microbe-associated molecular patterns that interferes with the infection process. Individual effectors are found in variable repertoires where some constituents target the same pathways. The effector protein AvrPto from *Pseudomonas syringae* has a core domain (CD) and C-terminal domain (CTD) that each promotes bacterial growth and virulence in tomato. The individual contributions of each domain and whether they act redundantly is unknown.

**Results:**

We use RNA-Seq to elucidate the contribution of the CD and CTD to the suppression of PTI in tomato leaves 6 h after inoculation. Unexpectedly, each domain alters transcript levels of essentially the same genes but to a different degree. This difference, when quantified, reveals that although targeting the same host genes, the two domains act synergistically. AvrPto has a relatively greater effect on genes whose expression is suppressed during PTI, and the effect on these genes appears to be diminished by saturation.

**Conclusions:**

RNA-Seq profiles can be used to observe relative contributions of effector subdomains to PTI suppression. Our analysis shows the CD and CTD multiplicatively affect the same gene transcript levels with a greater relative impact on genes whose expression is suppressed during PTI. The higher degree of up-regulation versus down-regulation during PTI is plausibly an evolutionary adaptation against effectors that target immune signaling.

**Electronic supplementary material:**

The online version of this article (doi:10.1186/s12864-016-2534-4) contains supplementary material, which is available to authorized users.

## Background

Plants are regularly exposed to pathogens and have evolved complex immune responses to combat them. In one response, plants detect microbe-associated molecular patterns (MAMPs), signatures of potentially pathogenic microbes, and activate pattern-triggered immunity (PTI) [1–3]. PTI is associated with the generation of reactive oxygen species, activation of MAP kinase cascades, callose deposition at the cell wall, and extensive transcriptional reprogramming [4–7]. Collectively, these responses prevent the deployment of some pathogenicity factors, suppress bacterial growth, and communicate the presence of a microbial threat to neighboring tissues [8, 9].

Plants use single-pass transmembrane extracellular pattern recognition receptors (PRRs) to detect MAMPs [10]. For example, in the interaction between tomato and the bacterial pathogen *Pseudomonas syringae* pv. *tomato,* the PRRs FLS2 and Bti9 activate PTI in response to the flagellin epitope flg22 and an unknown MAMP, respectively [11–14]. Another epitope of flagellin, flgII-28, also triggers PTI in tomato and has a major effect on the immunity-associated transcriptome [4, 15, 16].

Pathogens have evolved sophisticated systems to disarm and overcome PTI [1, 17]. *P. syringae* pv. *tomato* DC3000 is a widely-used model bacterial pathogen that utilizes a type III secretion system (T3SS) to translocate effector proteins from its cytoplasm to the host cytoplasm [18, 19]. Likely because of the complex nature of plant immune signaling, DC3000, other *P. syringae* strains and plant pathogens in related genera use highly variable type III effector (T3E) arsenals; DC3000, for example, is known to express and translocate at least 29 effector proteins [20, 21]. Several of these effector proteins target host complexes with known functions in immune signaling cascades [22, 23]. While several bacterial MAMPs are detected by tomato, flagellin has been shown to be the major elicitor of PTI in the interaction between DC3000 and tomato [4, 24].

Two DC3000 effector proteins, AvrPto and AvrPtoB, are especially effective at interfering with PTI [25]. An early study of their effect on gene expression using a tomato DNA microarray revealed they have partially overlapping effects and specifically induce genes associated with host ethylene production and response [26]. It is now known that each of these effectors targets the immune receptor complex containing FLS2 and a co-receptor BAK1 [27–29]. By doing so, they effectively suppress the expression of many of the host genes which are induced during PTI in response to flagellin; this subset of genes is referred to as flagellin-induced, repressed by effectors (FIRE genes) [4]. A DC3000Δ*avrPto*Δ*avrPtoB* mutant reaches lower population levels compared with wild type DC3000 in tomato leaves, and this reduced growth is alleviated upon one of two conditions: the re-introduction of either effector, or the deletion of the flagellin gene, *fliC* [30, 31]. Taken together, these observations indicate that AvrPto and AvrPtoB reduce the flagellin-induced PTI response by interfering with host transcriptional activation in response to FliC, and that this interruption of immune signaling is important for pathogen growth. The present study extends this research by investigating the contribution of specific domains of AvrPto to the suppression of the flagellin-induced transcriptome.

**Fig. 1:**
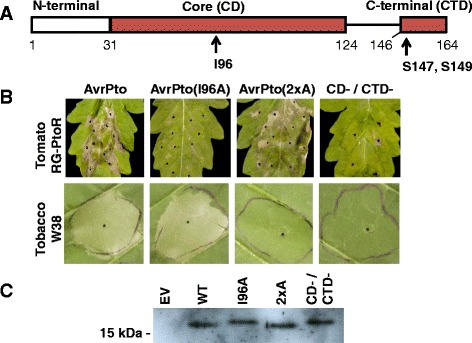
Delivery and expression of AvrPto and AvrPto variants. **a** A schematic of AvrPto showing the location of previously described alanine substitutions which specifically inactivate the functional domain they are in. **b** Hypersensitivity response in leaves of *S. lycopersicum* Rio Grande-PtoR (RG-PtoR) and *Nicotiana tabacum* L. c.v. W38 (W38). **c** Western blots showing protein abundance of AvrPto in D29E using a monoclonal anti-FLAG primary antibody with AvrPto proteins in part B modified to have a C-terminal FLAG epitope in the same plasmid backbone and with the same promoter. An anti-avrPto antibody used previously [31] detected non-specific proteins around 15 kDa, but gave similar results

AvrPto is an 18 kilodalton protein which contains two conserved virulence-promoting domains that have been shown to contribute multiplicatively to pathogen growth [32, 33]. One, hereafter referred to as the core domain (CD), spans amino acid residues 31 to 124 (Fig. 1a), and its crystal structure has been solved [34]. This domain interferes with the FLS2-BAK1 signaling complex directly [25]. In consequence, it inhibits mitogen-activated protein kinase (MAPK) signaling downstream of FLS2 and promotes pathogen growth; these activities are abolished in AvrPto proteins having a single amino acid substitution, isoleucine-96-alanine (I96A), in the CD [32]. MAPKs have been directly implicated in defense-related transcriptional control in Arabidopsis and tomato [35–37]. The second AvrPto domain, which is not part of the solved structure of AvrPto and which is dispensable for CD function is the C-terminal domain (CTD) that spans amino acid residues 146 to 164. The host target of this domain is unknown, but the domain is phosphorylated at several sites, with the phosphorylation of serine-147 and serine-149 being critical for promoting pathogen growth [38]. An AvrPto protein with alanine substitutions in both of these residues (S147A/S149A; referred to as 2xA) eliminates the contribution of this domain to pathogen growth during infection [32]. A major difference between the CD and the CTD is that only the former has been shown to inhibit MAPK signaling downstream of FLS2 [32].

Both domains are also associated with separate effector-triggered immunity (ETI) reactions. ETI is a more powerful defense response than PTI which includes localized programmed cell-death (PCD) in response to the detection of T3Es or their effects, or other pathogen molecules, by resistance (R) proteins [39]. This response effectively stops the pathogen at the expense of host tissue, and because of its severe phenotype of tissue collapse, can be useful for demonstrating effector delivery or function. The I96A substitution in the CD eliminates the ability of the host R proteins Pto kinase and Prf to coordinate and cause ETI [40]. Similar to I96A, the 2xA mutation of the CTD abolishes activation of an ETI response, but in tobacco instead of tomato and conferred by the putative R protein Rpa, that is distinct from Pto [32, 41]. This observation means that the two domains are recognized differentially by different protein-protein interactions in nature, thus further strengthening the notion that they behave like separate entities.

RNA-Seq is a powerful approach for monitoring transcriptome changes in response to stimuli [42–44]. The method is used to discover changes in transcript abundance that occur in specific developmental states and responses to various stimuli. Recently, RNA-Seq has been used to determine the transcriptional reprogramming that occurs in plant cells in response to PTI and ETI, and the changes to the PTI-induced transcriptome that bacteria make to facilitate infection [4, 6]. Here, we take advantage of a recently developed DC3000 strain, D29E, which has all known T3E-expressing genes deleted [45], to determine the individual contributions of the AvrPto CD and CTD on the host transcriptome at an early stage of the infection process.

## Results

### Use of the effector-deficient *Pst* strain D29E for monitoring AvrPto effects on the host transcriptome

To determine whether the activities of the AvrPto CD and CTD can be observed using strain D29E, we infiltrated D29E bacteria expressing plasmid-encoded *avrPto* into speck-resistant tomato leaves expressing *Pto* or into tobacco leaves expressing *Rpa*. We observed ETI-associated PCD in *Pto*-expressing tomato when the CD was unaltered and in tobacco when the CTD was unaltered (Fig. 1b). Forms of AvrPto with a substitution in the CD (I96A) or the CTD (2xA) did not elicit PCD in tomato or tobacco, respectively. An AvrPto variant carrying both CD and CTD substitutions mentioned above, hereafter referred to as CD^−^/CTD^−^, was unable to elicit PCD in leaves of either plant species. Equal amounts of AvrPto and the variants were expressed in D29E, indicating that the differences in these reactions are not due to a deficiency in effector abundance (Fig. 1c). The same constructs used in this study had been previously observed to be both produced and secreted to similar levels *in vitro* [32].

To examine the impact of the two domains on the transcriptome in susceptible tomato leaves we infiltrated the *P. syringae* strains expressing one of the four variants of AvrPto individually into tomato leaves lacking a functional Prf, Rio Grande-*prf3* (RG-*prf3*), and harvested tissue 6 h later. The strains did not exhibit differential growth at this timepoint (Additional file 1: Figure S1). The CD^−^/CTD^−^ variant was used as a negative control for domain activity as it does not affect PTI responses associated with AvrPto and does not contribute to pathogen growth in RG-*prf3* tomatoes. Four independent replicated experiments were performed and cDNA libraries were developed from each for RNA-Seq analysis (Table 1). Initial analyses of the data showed clear similarities among the four replicates within treatments and similar quality of all the libraries (Additional file 2: Table S1). Therefore, data from all four replicates were used for subsequent analysis.

**Table 1:** Treatments used for RNA-Seq analysis of PTI suppression by physical domains of AvrPto

Treatment	Concentration	Comment	Average total reads (in millions)	Average reads mapped (in percent)
Mock	10 mM MgCl_2_, 0.02 % silwet	No bacteria	15.2	93.1
D29E pCPP45::*avrPto*	OD_600_ 0.02	Wild-type	15.6	93.6
D29E pCPP45::*avrPto*(I96A)	OD_600_ 0.02	Core domain (CD) inactivated	16.3	94.1
D29E pCPP45::*avrPto*(2xA)^a^	OD_600_ 0.02	C-terminal domain (CTD) inactivated	16.6	94.3
D29E pCPP45::*avrPto*(I96A, 2xA)^a^	OD_600_ 0.02	‘CD^−^/CTD^−^‘	14.9	93.8

^a^2xA represents the presence of both S147A and S149A

### D29E induces a PTI response that is suppressed by AvrPto

**Fig. 2:**
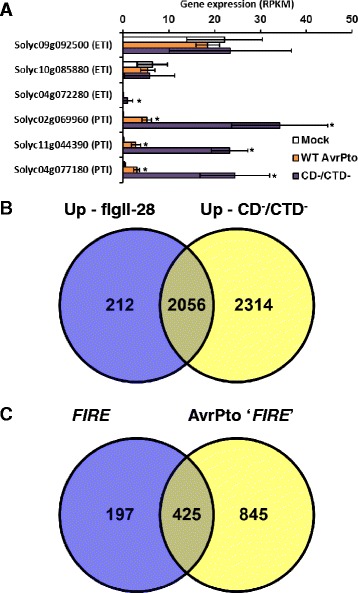
D29E induces PTI-associated gene expression that is suppressed by AvrPto. **a** Expression data for ETI- and PTI-specific marker genes described previously [6]. Gene expression values are RPKM averages of the four independent trials, and the error is one standard deviation based on those four replicate experiments. An asterisk indicates that the gene expression level is significantly different from a mock inoculation (p < 0.05) based on p-values corrected for multiple testing using the false discovery rate. **b** Venn diagram showing the number of genes whose transcript abundance is significantly increased in response to flgII-28 or D29E expressing CTD^−^/CD^−^, or both [4]. The criteria used are a positive-fold change of 2 or greater, an average RPKM of 3 or greater in at least one treatment, and a p-value of 0.05 or less. These criteria are the same used previously [4]. **c** Venn diagram including genes either meeting the criteria for a *FIRE* gene as described previously, or induced by D29E expressing CTD^−^/CD^−^ and then relatively repressed by D29E expressing AvrPto using the same statistical criteria as for the *FIRE* genes [4]

We first verified that our transcriptome profiles reflected induction of PTI and not ETI. For this, we used a set of 6 marker genes identified previously [46] whose expression is induced specifically during PTI or ETI. Analysis of these marker genes indicated that D29E inoculation induced PTI only, and that the presence of wild type AvrPto suppressed the expression of PTI-associated genes (Fig. 2a). There was a small, statistically significant increase in the ETI associated gene Solyc04g072280 for D29E delivering the CD^−^/CTD^−^, but the RPKM levels used in the comparison are either low or zero, WT AvrPto does not stimulate any differential response for this gene, and the other two ETI marker genes clearly show no ETI induction.

We next investigated whether the RNA-Seq profiles indicated D29E delivering the CD^−^/CTD^−^ activated PTI more broadly and whether wild type (WT) AvrPto generally suppressed the expression of PTI induced genes. A previous RNA-Seq analysis of the 6 h post-inoculation PTI response used the following criteria to limit the number of analyzed genes, and we refer to these genes as being ‘strongly up-regulated’, or ‘strongly down-regulated’: a) a two-fold or greater change in transcript abundance, up or down, measured in reads per kilobase per million (RPKM), b) a p-value corrected for multiple testing using the false discovery rate less than 0.05, and c) an average of 3 RPKM or greater in at least one treatment [4]. Using these criteria, we observed that over 90 % of the genes strongly up-regulated in the previous study by flgII-28, a flagellar PAMP, were strongly up-regulated by D29E delivering the inactivated AvrPto, showing that, as expected, PTI is strongly induced in the new transcriptome (Fig. 2b, Additional files 3 and 4).

Another 2314 genes were strongly up-regulated by D29E in addition to those previously found to be induced in response to flgII-28 [4]. In our earlier study, flgII-28 induced a similar number of genes as non-pathogenic bacteria, and those bacteria were infiltrated using a higher titer than in the present study (approximately10 times the CFU/mL compared to that used here). The additional induced genes we observed are likely due to either the higher statistical power because of our use of four replicates, the inoculation method used (syringe infiltration used previously versus vacuum infiltration here), or to unique characteristics of D29E, a highly modified pathogen, instead of a non-pathogen.

A previous study also defined a set of genes that are flagellin-induced, repressed by effectors (FIRE genes; [4]). We expected that a similar set of genes might be found by taking the set of genes that are strongly up-regulated during infection with D29E and asking which of these genes are down-regulated in the presence of AvrPto. Approximately 70 % of the original *FIRE* genes were found to be AvrPto-associated *FIRE* genes (Fig. 2c, Additional file 3). The original *FIRE* gene set also includes activity from AvrPtoB, another type III effector, and this additional activity might account for the remaining genes. The AvrPto-associated *FIRE* genes from the new set in this study also include 845 additional genes not included in the original *FIRE* set, perhaps a result of the increased sensitivity seen in the comparison between flgII-28 and D29E; if more PTI-associated genes are strongly up-regulated in the new transcriptome, more PTI-associated genes can be brought back below the threshold for strong induction by effector activity.

### The CD and CTD of AvrPto affect expression of the same host genes

**Fig. 3:**
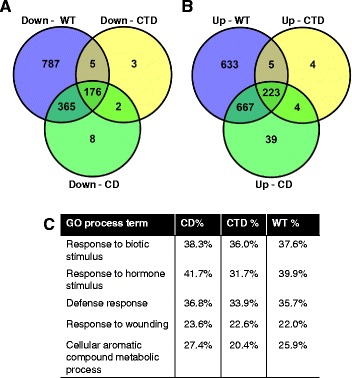
The C-terminal domain regulates a subset of the genes regulated by the core domain. **a** Venn diagram showing the number of genes that are significantly down-regulated, relative to D29E expressing the CD^−^/CTD^−^, by D29E expressing AvrPto (WT), AvrPto(2xA) (CD), or AvrPto(I96A) (CTD). **b** Venn diagram including the number of genes that are significantly up-regulated. **c** The top five defense-related gene ontology (GO) terms called from the set of genes down-regulated by AvrPto relative to the CD^−^/CTD^−^, calculated using the ‘GO term enrichment analysis’ tool with the False Discovery Rate metric [59]. Shown are the percentages of genes belonging to each GO term out of the total genes in that set for those significantly down-regulated by the each domain or the WT. Adjusted p-values for all GO terms shown are below 0.05 for all treatments

Using the same criteria for strong induction described above, genes were identified that are strongly up- or down-regulated by AvrPto, the CD or the CTD relative to the CD^−^/CTD^−^. As expected, almost all genes that are strongly up- or down-regulated by either domain are similarly regulated by the WT (Fig. 3a and b, Additional file 3). The CTD and CD also show a similar but unexpected pattern, with 96 % of genes that are strongly up- or down-regulated by the CTD of AvrPto are also strongly up- or down-regulated by the CD, respectively. However, for both up- and down-regulated genes, the CD affected about three-fold and two-fold more genes that meet our cutoff for strong regulation, respectively. To investigate if this difference between the domains might be due to different signaling pathways being affected, a GO term analysis of genes down-regulated by the WT, CD, and CTD was performed. Virtually the same statistically overrepresented GO terms were found to be associated with each of these domains at similar rates, providing evidence that the two domains affect expression of essentially the same host genes (Fig. 3c, Additional file 5). Thus, we surmised that the same signaling pathways are affected by both domains, but that these domains might additively contribute to PTI suppression.

### The CD affects expression of the same host genes as the CTD but to a greater degree

Because of the highly similar nature of our gene sets, we hypothesized that the additional genes affected by the CD compared to the CTD are an effect of more genes meeting the statistical cutoffs for strong induction, and not because of an additional set of genes being separately regulated by the CD. We therefore wished to analyze the relative induction strength of the CD compared to the CTD for all genes significantly regulated by either domain. To perform this analysis, it was necessary to use a method that compares the relative induction values of all genes that meet our criteria for strong induction as a group, thus allowing us to see if there is a trend in induction values close to our cutoff of two-fold change where the CD meets the cutoff, but not the CTD.

**Fig. 4:**
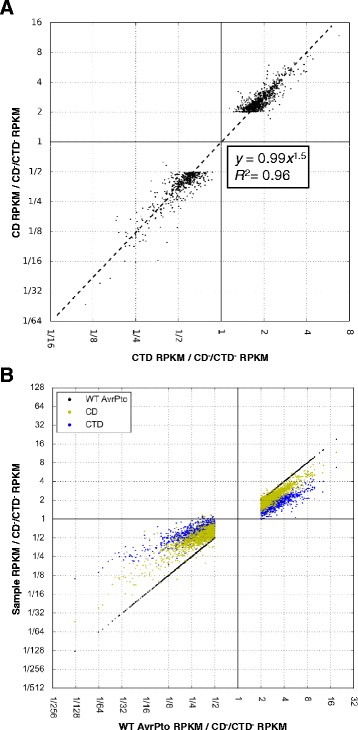
The quantitative relationship between the C-terminal and core domain gene induction for significantly regulated genes. **a** Scatterplot where each gene strongly up- or down-regulated by either domain relative to the CTD^−^/CD^−^ is represented by a single point with the induction values serving as coordinates (see Methods for criteria). The regression line and its r-squared value are calculated from a linear regression analysis performed on a log base 2 transformed set (a log transformed set is comprised of the logarithms of the original values) of the genes shown. **b** Scatterplot where the X coordinate equals induction by WT AvrPto relative to the CTD^−^/CD^−^, showing the induction ability of each domain versus the WT. The genes plotted are the set significantly up- or down- regulated by WT AvrPto, each gene is plotted 3 times with the same X coordinate and a variable Y coordinate based on identity, indicated by color

A method was therefore devised where induction ratios were plotted as coordinates on a two dimensional chart with logarithmic axes (see Methods for details). A strong correlation was found between the induction values relative to the CD^−^/CTD^−^ mutant for the set of genes that meet the criteria for significant regulation changes for either domain (Fig. 4a). A simple power function of y = 0.99x^1.5^, where x is the induction by the CTD and y is the induction by the CD, was derived from a linear regression analysis performed on the inductions when the logarithm is taken of each coordinate, also known as a log transformation (see Methods). This regression has an *R*^2^ value of 0.96 for the log base 2 transformed data set without further modification, indicating a strong correlation. This correlation is true for both up- and down-regulated genes, showing that the CD is more potent to the same quantitative level than the CTD for all genes showing strong regulation in either of these sets. Therefore, the CD and CTD affect the same genes, but the CD shows a greater effect in this dataset.

This same trend that was observed between the CD and CTD was also observed between WT AvrPto and the individual domains (Fig. 3a and b). To test whether reduced induction values are a major reason why fewer genes meet the statistical cutoff for the individual domains compared to the WT, the genes up- and down- regulated by the WT protein were plotted on a two dimensional chart (Fig. 4b). Each gene is plotted three times for comparison, one for each of WT, CD and CTD induction values. The induction values are all relative to the CD^−^/CTD^-^ mutant. Here, both domains up- and down-regulate in the same direction as the WT, but to a generally lower degree. The CD is generally closer than the CTD to the WT in induction, although the resolution between the CD and CTD is reduced at lower WT induction values. Therefore, WT AvrPto, the CD, and the CTD each affect expression of the same genes but with different potencies.

### The product of the inductions by the CD and CTD approximates the WT induction for down-regulated genes

Since both the CD and CTD of AvrPto regulate genes as if they are weaker versions of the WT, we hypothesized that the addition of inductions of the two domains might approximate the WT induction value. Since the domains’ induction values relative to the CD^−^/CTD^−^ have a linear relationship when plotted in a logarithmic chart, we propose that log[CD] + log[CTD] is the most appropriate method for combining their induction values, which can be re-written log[CD*CTD] (see Methods).

**Fig. 5:**
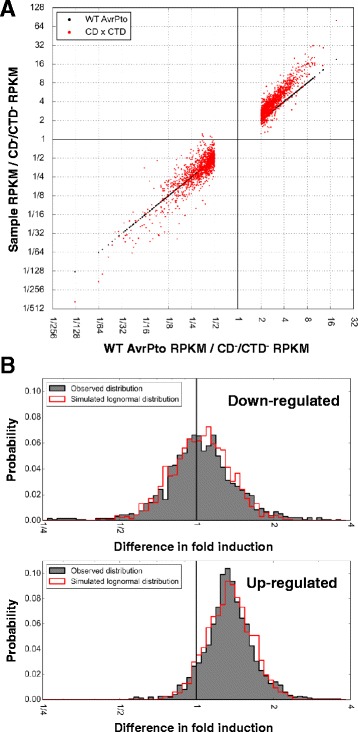
The product of inductions from the C-terminal and core domains potentially reveals WT saturation. **a** Scatterplot where the horizontal coordinate equals induction by WT AvrPto relative to the CTD^−^/CD^−^ and includes all genes strongly up- or down-regulated by AvrPto. The product of the gene inductions by the CD and CTD was calculated by multiplying the inductions relative to the CTD^−^/CD^−^. Each gene is plotted 2 times with the same X coordinate and a variable Y coordinate based on identity, indicated by color. **b** Histograms of residual values of WT compared to the CD^−^/CTD^−^ compared to the model combining the effects of both domains (gray). Histograms are binned according to value in 2^(1/16) sized bins. A simulated random lognormal distribution is shown in red, and contains the same number of data points as the set plotted from the transcriptome. The sets plotted are either those in Fig. 5a down-regulated (upper) or up-regulated (lower) by AvrPto. The sum of bin heights has been set equal to 1. Probability plots are included in Additional file 7: Figure S3 for both normal and log-normal distributions

When the induction values for the CD and CTD are multiplied together and plotted against the WT value for each gene using logarithmic axes, the values for the wild type protein seem to approximate the average value of the simple combination of induction for each domain, but only for genes down-regulated by WT AvrPto (Fig. 5a). A local average (includes neighboring genes in both the up- and down-regulation direction) of each product of the two domains for genes down-regulated by WT AvrPto returns an average value close to the WT for all frames, demonstrating that the average product of the CD and CTD induction values is close to the WT (Additional file 6: Figure S2). However, the up-regulated induction values of the WT are not approximated by the product of induction values of each domain; instead, the product overestimates the WT considerably and with increased severity for higher WT gene induction values (Fig. 5a).

This inconsistency between the up- and down-regulated genes is surprising since the individual domains appear to fit a single regression. One common test to show if a regression or model is appropriate is to look at the distribution of differences between the model and an observation, commonly referred to as residuals. If the differences are normally distributed, colloquially known as a Bell curve where two standard deviations account for 95 % of observations, or lognormal in the case of logarithmically distributed data, it is evidence for the validity of the model. We simulated a lognormal distribution for the up- and down-regulated sets of the data using the standard deviation and median values for the underlying normal function (statistics done on the log transformed data set), and used the same number of data points for each set to also get a sense for typical variance with our data sets up- or down-regulated by WT (*n* = 1471 and 1273, respectively) (Fig. 5b). For the set strongly up-regulated by WT, when non-log transformed, the median is 1.34 fold and a standard deviation is 1.23 fold. For the set strongly down-regulated by WT, they are 1.03 fold and 1.30 fold, respectively. The higher median value for the up-regulated set reflects the overestimation of the model, where the median value of approximately 1 for the down-regulated set reflects its accuracy. Both histograms show a distribution consistent with a lognormal distribution for the difference between the simple model and the WT. In the up-regulated genes there is a slight over-abundance of genes in the middle of the curve, and a mild skew towards higher values. Additionally, we have included a probability plot analysis which supports the interpretation here (Additional file 7: Figure S3). We were not able to determine from this dataset if the CD or CTD contributed more to the difference seen between WT and the model for the up-regulated genes. Overall, these observations are consistent with the method of analyzing relative induction values logarithmically, and suggest the difference between the model and observation may be naturally occurring and not an artifact. We therefore searched for a possible cause of the asymmetry in Fig. 5a.

### AvrPto has a greater proportional impact on genes that are down-regulated during PTI

We hypothesized that the overestimation by the simple multiplicative model seen in Fig. 5a of genes up-regulated by WT AvrPto reflects a saturation effect (where the values are influenced by a theoretical limit they cannot exceed). This hypothesis is based on Fig. 4a where the up- and down-regulated gene sets for the CD and CTD follow the same trend regardless of the direction of regulation, up or down. A saturation effect for only up-regulated genes could potentially address the imbalance seen between the up- and down-regulated genes seen in the WT, but not the domains individually.

At the early time point 6 h, it was shown previously [4] that during a successful infection with DC3000 the number of genes that meet the criteria for up- and down-regulation is decreased relative to a non-pathogen, and also shows more than twice the number of genes that are up-regulated compared to down-regulated. This supports the idea that effector proteins in DC3000, including AvrPto, alter the transcriptome to appear closer to a mock inoculation state, and also that PTI up-regulates genes more strongly than it down-regulates genes. We hypothesized, specifically, that the mock inoculation state represents a theoretical limit for transcript level modifications for effector proteins which block a signal that activates defense signaling.

**Fig. 6:**
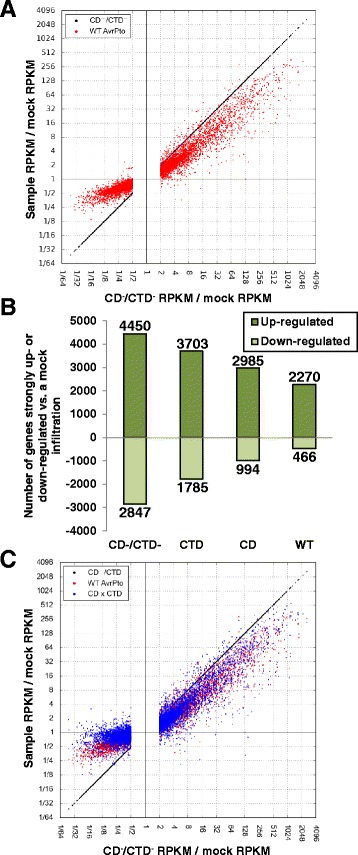
AvrPto has a proportionally greater effect on genes down-regulated during PTI. **a** Scatterplot where the horizontal coordinate equals induction by D29E delivering the CTD^−^/CD^−^ relative to a mock inoculation without bacteria, representing the PTI-induced transcriptome. Included are all genes significantly up- or down-regulated by PTI in this experimental setup. In red are the values for the same genes when WT AvrPto is delivered. **b** The number of genes with significantly altered transcript abundance compared to a mock infiltration for the CTD^−^/CD^−^, CTD, CD and WT. Above the axis is the number of genes that are strongly up-regulated, and below is the number of genes that are strongly down-regulated. The number of genes is written adjacent to the bar. **c** Scatterplot with all genes represented in Fig. 6a plotted with the same horizontal coordinate. Each gene is represented three times. In red are WT AvrPto modifications relative to a mock infection. In blue are the same genes as red except the reductions in PTI induction from each domain has been applied similarly to Fig. 5a. Note that the members lower left quadrants from Fig. 5a are now in the upper right quadrant here, and those in the upper right in Fig. 5a are now in the lower left

To test our hypothesis, we used the same plotting strategy, but used a comparison of the induction values of D29E delivering the CD^−^/CTD^−^ mutant compared to a mock inoculation (representing PTI induction), then compared these to D29E delivering WT AvrPto compared to a mock inoculation (Fig. 6a, Additional file 8: Figure S4). These show that for nearly all genes significantly up- or down-regulated by PTI, AvrPto brings their induction value closer to a mock inoculation value. AvrPto seems to target mostly, if not totally exclusively, genes whose expression levels are modified by PTI. A difference can be seen between the genes that are up- and down-regulated here: for PTI up-regulated genes, the average slope of the AvrPto-modified induction values appears to be closer to PTI (slope = 1) than for the down-regulated genes, where the change in apparent slope is more severe. This difference in shape shows that AvrPto has a proportionally greater effect up-regulating genes that are down-regulated during PTI.

In support of the saturation hypothesis are the number of genes that meet the statistical criteria for strong induction with and without CD and/or CTD activity (Fig. 6b, Additional file 9). Relative to a mock inoculation, D29E delivering the CD^−^/CTD^−^ mutant up-regulates 4450 genes, 2270 (51 %) of which are still significantly up-regulated with AvrPto activity. However, there are 2847 genes belonging to the corresponding down-regulated set, of which only 466 (16 %) are still down-regulated upon delivery of AvrPto. The down-regulated set of genes in this case is made of genes that are up-regulated by AvrPto relative to the CD^−^/CTD^−^ mutant, the same set where we see a potential saturation effect. This ability of AvrPto to bring over 80 % of the genes below our standard for strong regulation supports our hypothesis.

If saturation is preventing AvrPto from further up-regulating genes that are down-regulated by PTI, and the limit of transcriptional modifications by AvrPto result is the mock-inoculation state, then we should see few or no genes that are down-regulated during PTI increased past a mock- inoculation state when AvrPto is delivered. In Fig. 6a only a few PTI down-regulated genes have their transcript levels modified above that of a mock state by AvrPto. However, the combined effect of the two domains relative to a mock infiltration, performed similarly to Fig. 5a, where the differences relative to the CD^−^/CTD^−^ are multiplied, would result in 20 % of the genes down-regulated by PTI (induction 0.5 or less) being instead up-regulated compared to a mock state (Fig. 6c). A similar pattern is not found for the genes up-regulated by PTI, which instead seem to have the same general pattern in degree and variance for the multiplied product of the relative induction values for the CD and CTD.

The genes that are down-regulated during PTI are the same genes that are up-regulated by AvrPto relative to the CD^−^/CTD^−^ variant, meaning that for the same genes for which we observe a putative saturation effect of the WT induction values relative to the CD^−^/CTD^−^ via the multiplication of the two domains’ induction values, we also observe a putative saturation effect of the WT induction values relative to a mock infiltration. We conclude that our data set is consistent with the hypothesis that a mock inoculation serves as a theoretical limit for AvrPto modifications to the PTI-induced transcriptome at 6 h, that PTI proportionally more strongly up-regulates than down-regulates genes in response to DC3000 PAMPs, and that the modifications to the PTI-induced transcriptome by WT AvrPto in this dataset are saturated only for genes down-regulated during PTI. Therefore, AvrPto has a stronger proportional effect on genes down-regulated during PTI, and the genes up-regulated during PTI are more resistant to effector mediated modifications of the PTI-induced transcriptome.

## Discussion

We used the recently developed DC3000 derivative strain D29E to investigate how the CD and CTD affect host gene expression at an early stage of the infection process. Previously we had used a DC3000 strain having deletions in both *avrPto* and *avrPtoB* to study the combined effect of these effectors, but the 27 other actively-delivered effectors of this strain could obscure host responses as other effectors have been shown to target PTI signaling complex proteins or downstream signaling [1]. Supporting this idea, we found more genes affected by a single domain of AvrPto here than by the deletion of two effector genes, including *avrPto*. We did notice, however, that D29E is a stronger inducer of PTI than even non-pathogenic *Pseudomonas* strains, and it could be that some activity of the T3SS is further inducing PTI.

The CD and CTD of AvrPto were found to contribute multiplicatively to the suppression of virtually the same PTI-induced genes. This is true even though the two domains are responsible for triggering separate hypersensitive responses putatively through different R genes, suggesting that they have distinct host virulence targets and mechanisms. Furthermore, only the CD has been reported to inhibit activation of an early MAPK cascade [32], which in theory might be expected to result in different gene induction profiles. However, the two domains have been reported to make similar, additive contributions to bacterial growth during infection of tomato without other apparent differences, which is consistent with our findings here [32].

Our initial analysis revealed that the CD strongly affected the expression of a set of genes in addition to those strongly regulated by the CTD. Similarly, the WT strongly affected the expression of a set of genes in addition to those regulated by the CD. The genes regulated by any form of AvrPto are associated with generally the same gene ontology terms, further suggesting that they are involved in the same regulatory pathways. A plausible explanation for this is that the CD and CTD affect the same genes but to different magnitudes, and achieve this by targeting the same signal-transduction apparatus but at different locations.

To quantify the difference in magnitude, we independently developed a simple regression analysis method, key elements of which were first developed in a theoretical paper and previously applied to microarray data, including the multiplying of relative induction values from partial stimuli [47]. We direct the reader to Konishi 2005 for a detailed theoretical basis of this method. The regression method enabled us to create a simple linear regression of log-transformed relative induction values for the two domains. Using this approach, we found that by adding together the log effects of the CD and CTD relative to the CD^−^/CTD^−^ mutant, the WT could be approximated for down-regulated genes. We then presented evidence that the deviation from this trend in the AvrPto up-regulated genes is potentially the result of a saturation effect, stemming from a natural imbalance in the magnitude by which genes are up- and down-regulated during PTI. Evidence for such an imbalance was seen in previous studies from our group [4, 6], but now we can assign a significance to it: the higher magnitude of up-regulation by PTI resists transcriptional suppression by AvrPto of this set of genes which include the components of defense considered most important for pathogen resistance. Multiple T3Es within a single effector repertoire often appear to redundantly target the same pathway, but the reason for this has remained unclear, though it is generally thought to either confer additional benefits, or that redundancy is somehow important though individual effectors may be sufficient [21, 22, 48]. The multiple T3Es that seem to target this pathway then have potentially evolved to repeatedly target the set of genes up-regulated during PTI, with their repeated disruptions providing additional necessary reductions to the PTI-modified transcriptome.

The host target of the CTD is unknown, but the CTD-modified transcriptome points towards a role in disrupting early signaling. The solved structure of AvrPto does not contain the region of the highly-unstructured CTD, and it is unknown if these domains interact physically. The data presented here are highly suggestive that these domains target the same pathway even though they seem to be physically, functionally and immunologically distinct. Furthermore, we find no evidence in this dataset for domain-specific effects on the transcriptome.

The hypothesis that the CD and CTD target the same pathway is attractive since the two domains are in the same polypeptide and simultaneously could disrupt PTI signaling at or just downstream of the BAK1/FLS2 complex. This is not necessarily the same as having the same mechanism. For example, if the CD blocks MAPK activation, while the CTD targets a signaling component for proteolysis, then similar changes to the transcriptome might be observed though the mechanisms are different. Investigations into whether the two domains interact with the same or different proteins in a PRR-containing protein complex would provide clarity to how these domains achieve similar profiles.

There are several effector proteins that are known to have separate physical domains with distinct properties. Two domains with different biological activities have been described for the Salmonella T3E SptP; the GTPase activating activity causes cytoskeletal rearrangements, while the activity of its tyrosine phosphatase has an unknown role in virulence promotion [49, 50]. *Xanthomonas* TALENs also have a modular structure, with separate domains for DNA-binding and RNA polymerase activating activities [51]. *Pseudomonas* effector AvrPtoB is a well-documented example of an effector protein that targets multiple plant immunity proteins [25]. Its N-terminal domain binds to and inhibits kinase activity of Bti9 a possible PRR, while a central domain binds to BAK1 and interferes with FLS2/BAK1 immune signaling [11, 12, 27]. Targeting of multiple immune complexes by separate domains within an effector protein is possibly what is occurring with the CD and CTD of AvrPto.

On a broader scale, the methodology devised here could be used to dissect whether or not two proteins affect the same signaling pathways and whether they have the same magnitude of impact on the transcriptome. It can also identify overlaps in function. Usually, and as we originally planned, RNA-Seq is used to identify individual genes within a transcriptome that differentiate two different stimuli. Here, we have instead used it to quantify the stimuli and describe the structure of the transcriptome. We suspect that similar methods could be used on existing data sets to uncover more features of the dynamic transcriptome.

## Conclusions

RNA-Seq is a powerful tool for investigating pathogenicity factors that target host immune signaling pathways, especially combined with pathogens modified to have reduced pathogenicity. As shown here, such analyses can reveal subtle differences in the contribution of pathogenicity factor protein domains to immune suppression. Two alanine-substitution mutants representing the inactivation of separate physical domains of the type III effector protein AvrPto are shown to affect the same genes, but to different degrees. When used to model the whole protein containing both active domains, a key difference between genes up- and down-regulated during pattern-triggered immunity is revealed. Genes are more strongly up-regulated than down-regulated during PTI, making up-regulated genes more resistant to muting by bacterially derived effectors.

## Methods

### Strains

D29E is the only bacterial strain used in this study [45]. pCPP45 and pDSK519 based vectors were transformed into D29E using standard electroporation protocols [52]. Details of the strains and plasmids used are presented in Additional file 10.

### Hypersensitive response

Bacteria suspended in 10 mM MgCl_2_ were syringe infiltrated into leaves of *Solanum lycopersicum* cv. Rio Grande-PtoR and *S. lycopersicum* cv. Rio Grande-*prf3* leaves at an OD_600_ of 0.02 and *Nicotiana tabacum* W38 at an OD_600_ of 0.2. Leaves were photographed 24–48 h after infiltration.

### Assays for expression

Expression of AvrPto in D28E, the parent of D29E, was assayed as described previously, except that the AvrPto molecules contained a C-terminal FLAG epitope peptide fusion, and this was detected using an anti-FLAG HRP-conjugate antibody (Sigma Aldrich A8592) [53].

### Pathogen inoculation

*S. lycopersicum* cv. Rio Grande-*prf3* plants were infiltrated approximately 3 weeks after transplanting between 9 and 11 am and harvested 6 h after inoculation. Inocula consisted of 0.02 % Silwet, 10 mM MgCl_2_, and bacteria resuspended at OD_600_ 0.02. The four side leaflets of the youngest fully expanded leaf were harvested, their main vein removed, and snap-frozen in liquid N_2_ with small steel balls for RNA extraction. Four independent replicate experiments were performed in four sequential weeks.

### RNA Extraction, cDNA library construction and RNA-Seq analysis

The RNA-Seq methods were performed exactly as described previously [6].

### Data analysis

Data analysis was performed using Microsoft Excel and the Enthrought Canopy Python distribution [54]. The modules SciPy, Ipython, MatPlotLib, and xlrd were the modules primarily used [55–58].

### Plots of relative induction values

For these genes, the base 2 logarithmic transformation is made of the induction values to aid human interpretation (the base 2 logarithm is made for each datum individually). A base 2 logarithmic axis is used accordingly. Each coordinate along the horizontal axis represents the relative induction value of a gene in the data set in order to make a comparison. Generally, this will be the data set that best represents the statistical cutoffs used for defining the set of genes plotted. The vertical coordinate(s) for each gene are then plotted in the same way, but several vertical coordinates may be used for the same gene.

### Regression analysis

A linear regression was performed on the base 2 log-transformed set of induction values for the genes plotted in Fig. 4a. The r^2^ value was derived from the fit of this line to the log-transformed data set. The regression formula is derived from the linear regression equation, rearranged from log_2_[y] = m • log_2_[x] + b.

### Moving average analysis

A program was created that takes an average of the vertical coordinate for each gene and the ten-gene window of the closest genes, both greater and less, in horizontal coordinate value. The average was then plotted for each gene. Similar results are achieved using greater or smaller window sizes and can be changed relatively easily in the program provided. The 21-gene window presented in Additional file 6 represents a balance for presentation between over-smoothing and noise.

### Ethics approval and consent to participate

Not applicable.

### Consent for publication

Not applicable.

### Availability of data and materials

The raw reads data is available at NCBI Sequence Read Archive (SRA) under accession SRP065499. Data from processed reads is available at the Tomato Functional Genomics Database (http://ted.bti.cornell.edu/) under accession D013 in ‘RNA-seq data’.

The Python computer code in IPython Notebook format used to analyze this data is available at https://github.com/jnw29/AvrPto_Transcriptome.

## Additional files

Additional file 1: Figure S1. D29E populations delivering any form of AvrPto are stable at 6hpi. Growth data for D29E strains delivering AvrPto 6 h after infiltrating using the same concentrations as in the samples prepared for RNA-Seq. The counts are an average of three independent experiments performed on sequential weeks with similar results. No significant growth or death is seen at 6 h. (PDF 198 kb) Additional file 2: Table S1. Read quality of the RNA-Seq reactions used in this study. (XLSX 12 kb) Additional file 3: Excel workbook. Lists of genes showing significant regulation in Figs. 2 and 3, including the criterion data. (XLS 1602 kb) Additional file 4: Excel workbook. Lists of genes meeting the criteria used in Rosli et al. 2013 in Fig. 2, including the criterion data. (XLSX 71 kb) Additional file 5: Excel workbook. Lists of gene ontology terms associated with each domain in Fig. 3. (XLSX 910 kb) Additional file 6: Figure S2. A 21-gene window moving average of the effect of the combined domains. A 21-gene window average (includes the gene at the X coordinate which represents a gene down-regulated by WT AvrPto plus 10 on either side) is shown for each gene in the set down-regulated by WT AvrPto relative to the CD^−^/CTD^−^ (blue line) except for the 10 genes with the highest and lowest inductions by WT AvrPto. The WT values are shown in black and the multiplied domains induction as transparent red for comparison. Each gene is represented 3 times except for the 20 mentioned previously not included in the moving average, those genes are represented twice. This set of averages centered on individual genes shows that the trend of the down-regulated by WT AvrPto gene set, arranged by WT-induction values, is roughly matched by the trend in the two domains combined. A linear regression of the log base 2-transformed set of the genes shown in the moving average against the WT AvrPto values returns values that translate into a power function regression of 1.1x0.97 with an r2 value of 0.97 (see script for additional details). This suggests that noise is a limiting factor in combining domain induction values together for individual genes, but the trends may be accurate with the proper reference. (PDF 153 kb) Additional file 7: Figure S3. Probability plot analysis on log-transformed relative induction models. Probability plots generated using SciPy module for Python showing normal distributions are more consistent with the log-transformed data sets. Plots A and B are made using log transformed data of the residuals (differences) between the multiplied model and WT data for the genes down- and up-regulated by AvrPto, respectively. The R2 values of A and B are 0.981 and 0.989, respectively. Both plots are heavy tailed and indicate some over-representation of values around the average compared to an ideal lognormal distribution. (PDF 51 kb) Additional file 8: Excel workbook. Lists of genes up- and down-regulated relative to a mock inoculation, used in Fig. 6b. (PDF 300 kb) Additional file 9: Figure S4. No group of genes in this transcriptome are seemingly regulated independently of PTI. A scatterplot where the X coordinate equals induction by D29E pCPP45::*avrPto*(I96A,2xA) relative to a mock inoculation without bacteria, representing the PTI-induced transcriptome. Only RPKM values were used to select this data set, and there is not a set of genes strongly up- or down-regulated by the CD or CTD combined independently of PTI. (XLS 2504 kb) Additional file 10: Table S2. Strains and plasmids used in this study. (XLSX 10 kb)

## Abbreviations

2xA: noth S147A and S149A
CD: AvrPto core domain
CD-/CTD: AvrPto(I96A, S147A, S149A)
CTD: AvrPto C-terminal domain
ETI: effector triggered immunity
FIRE: flagellin induced repressed by effectors
MAMP: microbe associated molecular pattern
MAPK: mitogen activated protein kinase
PRR: pattern recognition receptor
PTI: Pamp triggered immunity
R protein: resistance protein
RG-prf3: rio grande-prf3
RG-PtoR: rio grande-PtoR
RPKM: reads per kilobase per million mapped reads
T3E: type III effector
T3SS: type III secretion system
WT: AvrPto, wild type

## References

[1] Dodds PN, Rathjen JP (2010). Plant immunity: towards an integrated view of plant-pathogen interactions. Nat Rev Genet.

[2] Schwessinger B, Ronald PC (2012). Plant innate immunity: perception of conserved microbial signatures. Annual Review of Plant Biology.

[3] Segonzac C, Zipfel C (2011). Activation of plant pattern-recognition receptors by bacteria. Curr Opin Microbiol.

[4] Rosli HG, Zheng Y, Pombo MA, Zhong S, Bombarely A, Fei Z, Collmer A, Martin GB (2013). Transcriptomics-based screen for genes induced by flagellin and repressed by pathogen effectors identifies a cell wall-associated kinase involved in plant immunity. Genome Biol.

[5] Buscaill P, Rivas S (2014). Transcriptional control of plant defence responses. Curr Opin Plant Biol.

[6] Pombo MA, Zheng Y, Fernandez-Pozo N, Dunham DM, Fei Z, Martin GB (2014). Transcriptomic analysis reveals tomato genes whose expression is induced specifically during effector-triggered immunity and identifies the Epk1 protein kinase which is required for the host response to three bacterial effector proteins. Genome Biol.

[7] Glazebrook J (2005). Contrasting mechanisms of defense against biotrophic and necrotrophic pathogens. Annu Rev Phytopathol.

[8] Crabill E, Joe A, Block A, van Rooyen JM, Alfano JR (2010). Plant immunity directly or indirectly restricts the injection of type III effectors by the *Pseudomonas syringae* type III secretion system. Plant Physiol.

[9] Pieterse CM, Van der Does D, Zamioudis C, Leon-Reyes A, Van Wees SC (2012). Hormonal modulation of plant immunity. Annual Review of Cell and Developmental Biology.

[10] Zipfel C (2014). Plant pattern-recognition receptors. Trends Immunol.

[11] Zeng L, Velasquez AC, Munkvold KR, Zhang J, Martin GB (2012). A tomato LysM receptor-like kinase promotes immunity and its kinase activity is inhibited by AvrPtoB. Plant J.

[12] Cheng W, Munkvold KR, Gao H, Mathieu J, Schwizer S, Wang S, Yan YB, Wang J, Martin GB, Chai J (2011). Structural analysis of *Pseudomonas syringae* AvrPtoB bound to host BAK1 reveals two similar kinase-interacting domains in a type III effector. Cell Host Microbe.

[13] Robatzek S, Bittel P, Chinchilla D, Kochner P, Felix G, Shiu SH, Boller T (2007). Molecular identification and characterization of the tomato flagellin receptor LeFLS2, an orthologue of Arabidopsis FLS2 exhibiting characteristically different perception specificities. Plant Molecular Biology.

[14] Chakravarthy S, Velasquez AC, Ekengren SK, Collmer A, Martin GB (2010). Identification of *Nicotiana benthamiana* genes involved in pathogen-associated molecular pattern-triggered immunity. Mol Plant-Microbe Interact.

[15] Clarke CR, Chinchilla D, Hind SR, Taguchi F, Miki R, Ichinose Y, Martin GB, Leman S, Felix G, Vinatzer BA (2013). Allelic variation in two distinct *Pseudomonas syringae* flagellin epitopes modulates the strength of plant immune responses but not bacterial motility. New Phytol.

[16] Cai RM, Lewis J, Yan SC, Liu HJ, Clarke CR, Campanile F, Almeida NF, Studholme DJ, Lindeberg M, Schneider D (2011). The plant pathogen *Pseudomonas syringae* pv. *tomato* is genetically monomorphic and under strong selection to evade tomato immunity. PLoS Pathog.

[17] Wirthmueller L, Maqbool A, Banfield MJ (2013). On the front line: structural insights into plant-pathogen interactions. Nature Reviews Microbiology.

[18] Cuppels DA (1986). Generation and characterization of Tn5 insertion mutations in *Pseudomonas syringae* pv. *tomato*. Applied and Environmental Microbiology.

[19] Tampakaki AP, Skandalis N, Gazi AD, Bastaki MN, Panagiotis FS, Charova SN, Kokkinidis M, Panopoulos NJ (2010). Playing the “harp”: evolution of our understanding of *hrp/hrc* genes. Annual Review Phytopathology.

[20] Buell CR, Joardar V, Lindeberg M, Selengut J, Paulsen IT, Gwinn ML, Dodson RJ, Deboy RT, Durkin AS, Kolonay JF (2003). The complete genome sequence of the Arabidopsis and tomato pathogen *Pseudomonas syringae* pv. *tomato* DC3000. Proc Natl Acad Sci USA.

[21] Lindeberg M, Cunnac S, Collmer A (2012). *Pseudomonas syringae* type III effector repertoires: last words in endless arguments. Trends Microbiol.

[22] Feng F, Zhou JM (2012). Plant-bacterial pathogen interactions mediated by type III effectors. Curr Opin Plant Biol.

[23] Deslandes L, Rivas S (2012). Catch me if you can: bacterial effectors and plant targets. Trends Plant Sci.

[24] Felix G, Duran JD, Volko S, Boller T (1999). Plants have a sensitive perception system for the most conserved domain of bacterial flagellin. Plant J.

[25] Martin GB. Suppression and activation of the plant immune system by Pseudomonas syringae effectors AvrPto and AvrPtoB. In: Effectors in Plant-Microbe Interactions. Edited by Martin F, Kamoun S. Oxford, UK: Wiley-Blackwell; 2012. p. 123–154.

[26] Cohn JR, Martin GB (2005). *Pseudomonas syringae* pv. *tomato* type III effectors AvrPto and AvrPtoB promote ethylene-dependent cell death in tomato. Plant J.

[27] Shan L, He P, Li J, Heese A, Peck SC, Nurnberger T, Martin GB, Sheen J (2008). Bacterial effectors target the common signaling partner BAK1 to disrupt multiple MAMP receptor-signaling complexes and impede plant immunity. Cell Host Microbe.

[28] Gohre V, Spallek T, Haweker H, Mersmann S, Mentzel T, Boller T, de Torres M, Mansfield JW, Robatzek S (2008). Plant pattern-recognition receptor FLS2 is directed for degradation by the bacterial ubiquitin ligase AvrPtoB. Curr Biol.

[29] Kaushal P, Malaviya DR, Roy AK (2004). Prospects for breeding apomictic rice: a reassessment. Current Science.

[30] Lin NC, Martin GB (2005). An *avrPto/avrPtoB* mutant of *Pseudomonas syringae* pv. *tomato* DC3000 does not elicit Pto-mediated resistance and is less virulent on tomato. Mol Plant Microbe Interact.

[31] Kvitko BH, Park DH, Velasquez AC, Wei CF, Russell AB, Martin GB, Schneider DJ, Collmer A (2009). Deletions in the repertoire of *Pseudomonas syringae* pv. *tomato* DC3000 type III secretion effector genes reveal functional overlap among effectors. PLoS Pathog.

[32] Yeam I, Nguyen HP, Martin GB (2010). Phosphorylation of the *Pseudomonas syringae* effector AvrPto is required for FLS2/BAK1-independent virulence activity and recognition by tobacco. Plant J.

[33] Nguyen HP, Yeam I, Angot A, Martin GB (2010). Two virulence determinants of type III effector AvrPto are functionally conserved in diverse *Pseudomonas syringae* pathovars. New Phytol.

[34] Wulf J, Pascuzzi PE, Fahmy A, Martin GB, Nicholson LK (2004). The solution structure of type III effector protein AvrPto reveals conformational and dynamic features important for plant pathogenesis. Structure.

[35] Meng X, Zhang S (2013). MAPK cascades in plant disease resistance signaling. Annu Rev Phytopathol.

[36] Pedley KF, Martin GB (2005). Role of mitogen-activated protein kinases in plant immunity. Curr Opin Plant Biol.

[37] Ekengren SK, Liu Y, Schiff M, Dinesh-Kumar SP, Martin GB (2003). Two MAPK cascades, NPR1, and TGA transcription factors play a role in Pto-mediated disease resistance in tomato. Plant J.

[38] Anderson JC, Pascuzzi PE, Xiao F, Sessa G, Martin GB (2006). Host-mediated phosphorylation of type III effector AvrPto promotes *Pseudomonas* virulence and avirulence in tomato. Plant Cell.

[39] Cui H, Tsuda K, Parker JE (2014). Effector-triggered immunity: from pathogen perception to robust defense. Ann Rev Plant Biol.

[40] He P, Shan L, Lin NC, Martin GB, Kemmerling B, Nurnberger T, Sheen J (2006). Specific bacterial suppressors of MAMP signaling upstream of MAPKKK in Arabidopsis innate immunity. Cell.

[41] Shan L, Thara VK, Martin GB, Zhou JM, Tang X (2000). The *Pseudomonas* AvrPto protein is differentially recognized by tomato and tobacco and is localized to the plant plasma membrane. Plant Cell.

[42] Mutz KO, Heilkenbrinker A, Lonne M, Walter JG, Stahl F (2013). Transcriptome analysis using next-generation sequencing. Curr Opin Biotechnol.

[43] Martin LB, Fei Z, Giovannoni JJ, Rose JK (2013). Catalyzing plant science research with RNA-seq. Frontiers Plant Sci.

[44] Morozova O, Hirst M, Marra MA (2009). Applications of new sequencing technologies for transcriptome analysis. Annu Rev Genomics Hum Genet.

[45] Wei HL, Chakravarthy S, Mathieu J, Helmann TC, Stodghill P, Swingle B, Martin GB, Collmer A (2015). *Pseudomonas syringae* pv. *tomato* DC3000 type III secretion effector polymutants reveal an interplay between HopAD1 and AvrPtoB. Cell Host Microbe.

[46] Pombo MA, Zheng Y, Fernandez-Pozo N, Dunham DM, Fei Z, Martin GB: Transcriptomic analysis reveals tomato genes whose expression is induced specifically during effector-triggered immunity and identifies the Epk1 protein kinase which is required for the host response to two bacterial effector proteins. in review 201x. 10.1186/s13059-014-0492-1 PMC4223163 25323444

[47] Konishi T (2005). A thermodynamic model of transcriptome formation. Nucleic Acids Research.

[48] Block A, Alfano JR (2011). Plant targets for Pseudomonas syringae type III effectors: virulence targets or guarded decoys?. Curr Opin Microbiol.

[49] Fu Y, Galan JE (1999). A salmonella protein antagonizes Rac-1 and Cdc42 to mediate host-cell recovery after bacterial invasion. Nature.

[50] Humphreys D, Hume PJ, Koronakis V (2009). The Salmonella effector SptP dephosphorylates host AAA+ ATPase VCP to promote development of its Intracellular replicative niche. Cell Host Microbe.

[51] Doyle EL, Stoddard BL, Voytas DF, Bogdanove AJ (2013). TAL effectors: highly adaptable phytobacterial virulence factors and readily engineered DNA targeting proteins. Trends in Cell Biology.

[52] Choi KH, Kumar A, Schweizer HP (2006). A 10-min method for preparation of highly electrocompetent Pseudomonas aeruginosa cells: application for DNA fragment transfer between chromosomes and plasmid transformation. J Microbiol Methods.

[53] Cunnac S, Chakravarthy S, Kvitko BH, Russell AB, Martin GB, Collmer A (2011). Genetic disassembly and combinatorial reassembly identify a minimal functional repertoire of type III effectors in *Pseudomonas syringae*. Proc Natl Acad Sci USA.

[54] Enthought Python Distribution (Version 7.3). 2012. Retrieved from https://support.enthought.com/hc/en-us/articles/204468990-How-do-I-cite-Canopy-or-EPD-EnthoughtPython.

[55] Jones E, Oliphant E, Peterson, P, et al. SciPy: open source scientific tools for Python; 2001. http://www.scipy.org/. (Accessed 2014-03-01).

[56] Perez F, Granger BE (2007). IPython: A system for interactive scientific computing. Comput Sci Eng.

[57] Hunter JD (2007). Matplotlib: a 2D graphics environment. Computing in Science & Engineering.

[58] Machin J, Lingfo Pty Ltd.: xlrd. 2009.

[59] Boyle EI, Weng S, Gollub J, Jin H, Botstein D, Cherry JM, Sherlock G (2004). GO::TermFinder—open source software for accessing Gene Ontology information and finding significantly enriched Gene Ontology terms associated with a list of genes. Bioinformatics (Oxford, England).

