# Supplementary figures and images for: A novel method of transcriptome interpretation reveals a quantitative suppressive effect on tomato immune signaling by two domains in a single pathogen effector protein
Source: BMC Genomics. 2016 Mar 14;17:229. doi: 10.1186/s12864-016-2534-4 (PMC4790048; doi:10.1186/s12864-016-2534-4)

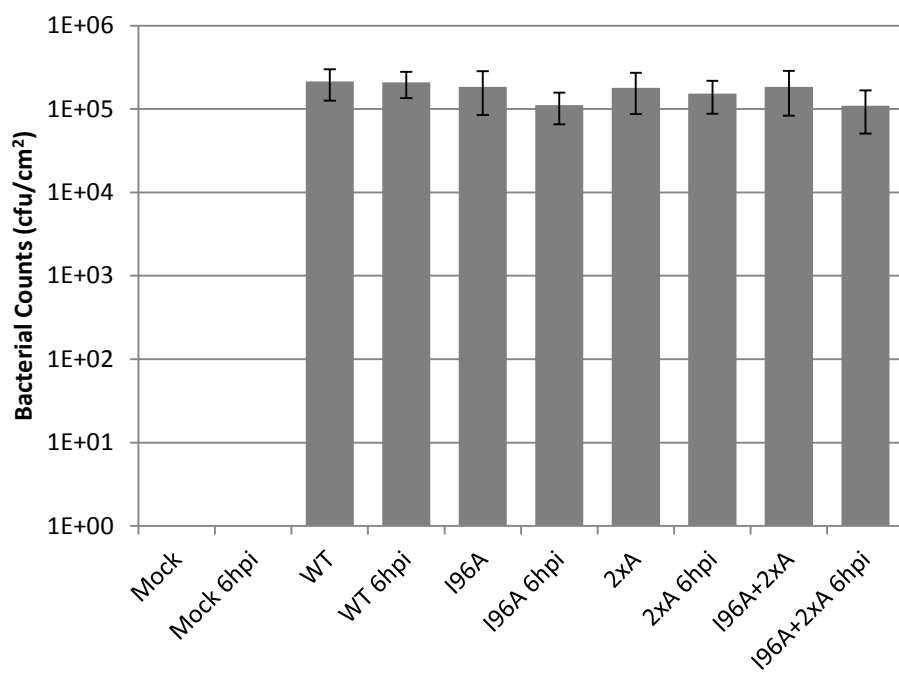

**Figure S1**

Supplement: Additional file 1: Figure S1. — D29E populations delivering any form of AvrPto are stable at 6hpi. Growth data for D29E strains delivering AvrPto 6 h after infiltrating using the same concentrations as in the samples prepared for RNA-Seq. The counts are an average of three independent experiments performed on sequential weeks with similar results. No significant growth or death is seen at 6 h. (PDF 198 kb) [file 12864_2016_2534_MOESM1_ESM.pdf]

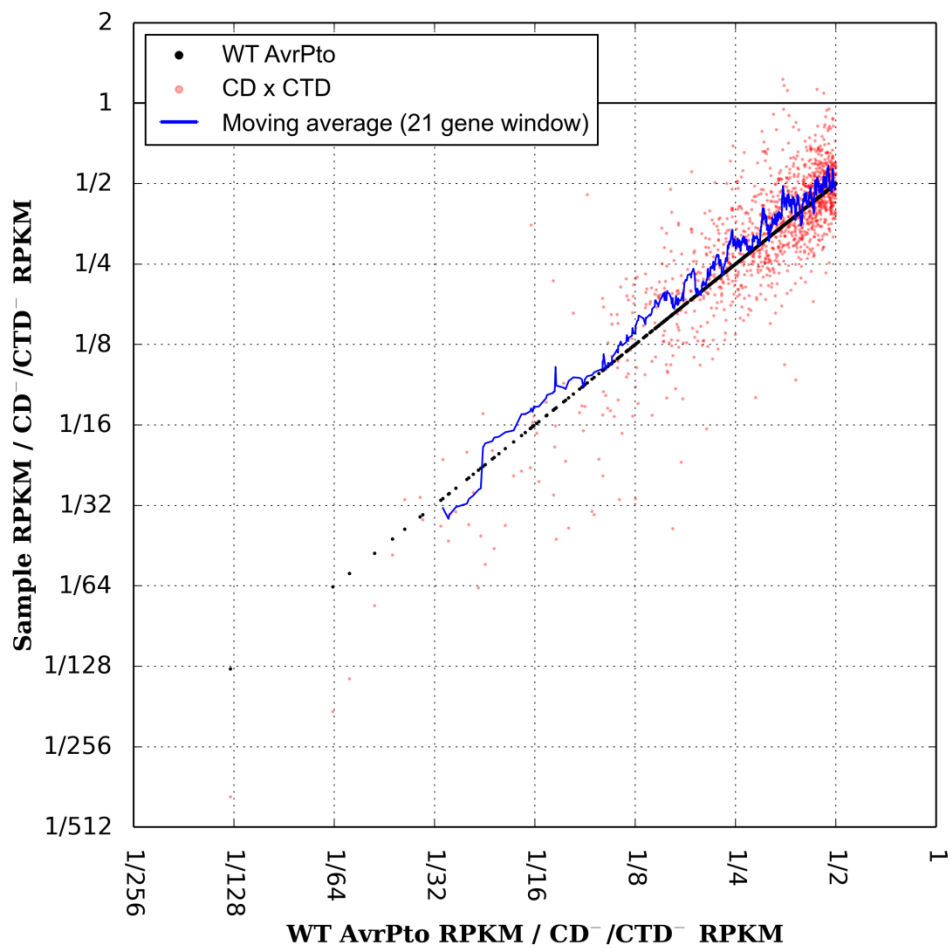

**Figure S2**

Supplement: Additional file 6: Figure S2. — A 21-gene window moving average of the effect of the combined domains. A 21-gene window average (includes the gene at the X coordinate which represents a gene down-regulated by WT AvrPto plus 10 on either side) is shown for each gene in the set down-regulated by WT AvrPto relative to the CD−/CTD− (blue line) except for the 10 genes with the highest and lowest inductions by WT AvrPto. The WT values are shown in black and the multiplied domains induction as transparent red for comparison. Each gene is represented 3 times except for the 20 mentioned previously not included in the moving average, those genes are represented twice. This set of averages centered on individual genes shows that the trend of the down-regulated by WT AvrPto gene set, arranged by WT-induction values, is roughly matched by the trend in the two domains combined. A linear regression of the log base 2-transformed set of the genes shown in the moving average against the WT AvrPto values returns values that translate into a power function regression of 1.1x0.97 with an r2 value of 0.97 (see script for additional details). This suggests that noise is a limiting factor in combining domain induction values together for individual genes, but the trends may be accurate with the proper reference. (PDF 153 kb) [file 12864_2016_2534_MOESM6_ESM.pdf]

**A**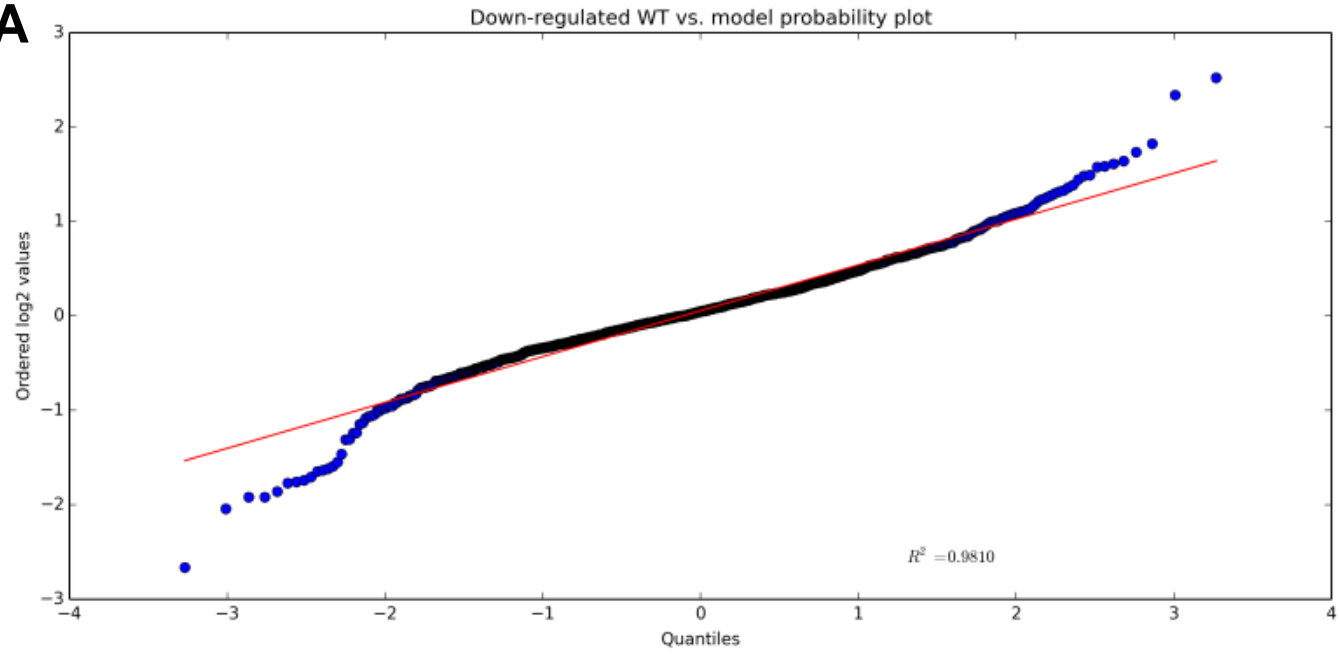**B**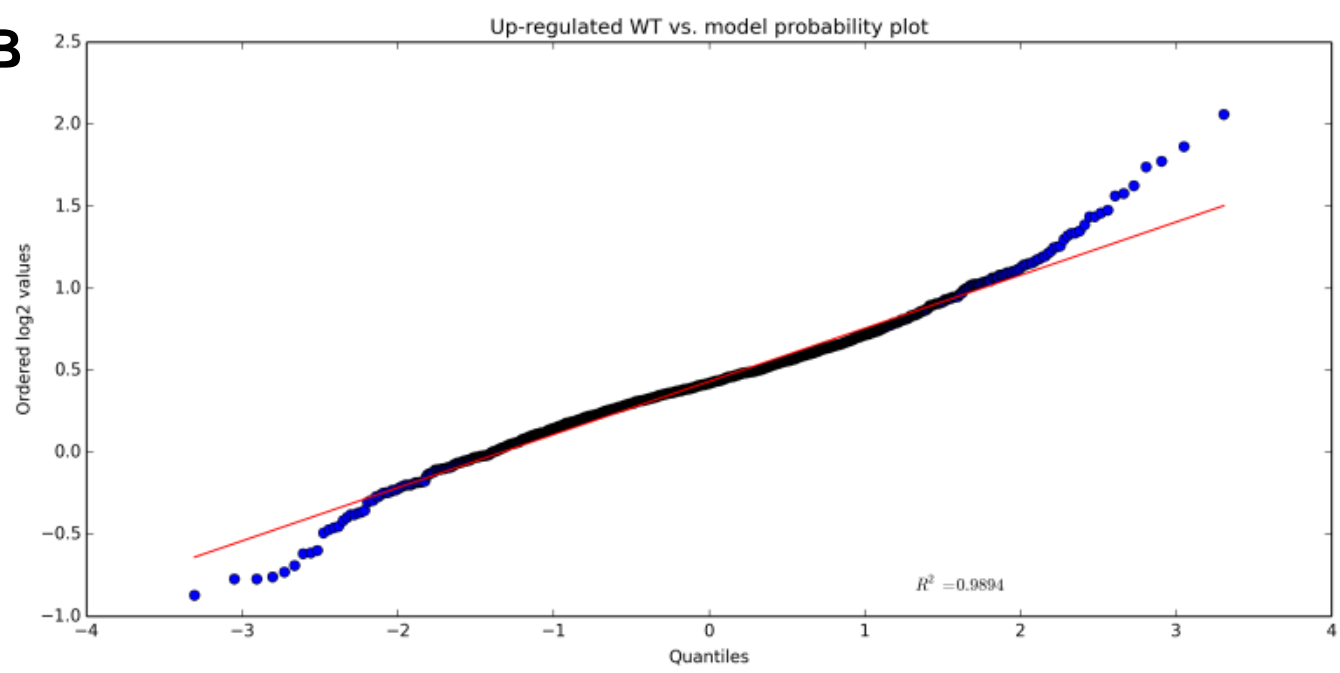**Figure S3**

Supplement: Additional file 7: Figure S3. — Probability plot analysis on log-transformed relative induction models. Probability plots generated using SciPy module for Python showing normal distributions are more consistent with the log-transformed data sets. Plots A and B are made using log transformed data of the residuals (differences) between the multiplied model and WT data for the genes down- and up-regulated by AvrPto, respectively. The R2 values of A and B are 0.981 and 0.989, respectively. Both plots are heavy tailed and indicate some over-representation of values around the average compared to an ideal lognormal distribution. (PDF 51 kb) [file 12864_2016_2534_MOESM7_ESM.pdf]

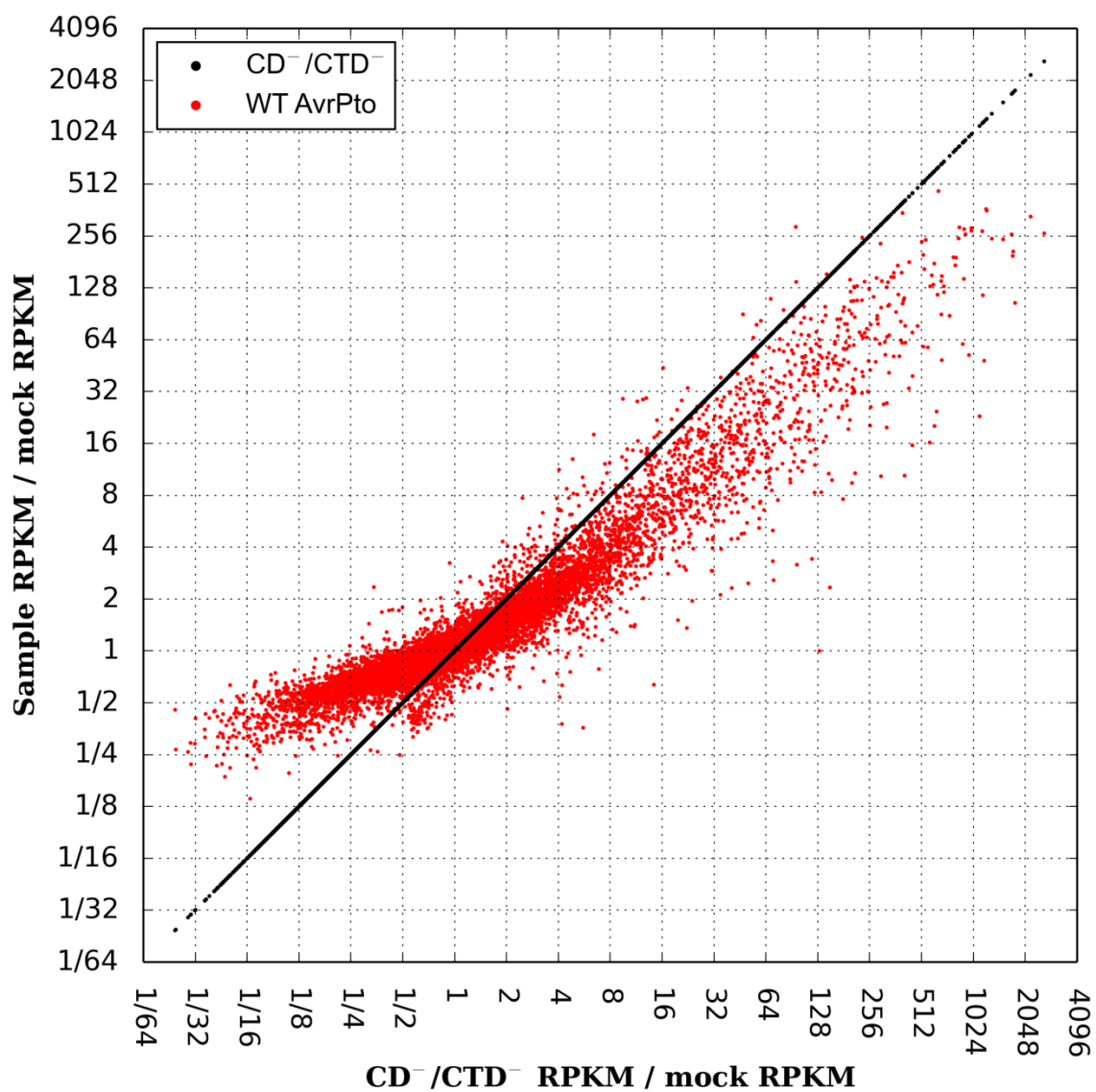

**Figure S4**

Supplement: Additional file 8: — Excel workbook. Lists of genes up- and down-regulated relative to a mock inoculation, used in Fig. 6b. (PDF 300 kb) [file 12864_2016_2534_MOESM8_ESM.pdf]
